# Mutual Information between Discrete and Continuous Data Sets

**DOI:** 10.1371/journal.pone.0087357

**Published:** 2014-02-19

**Authors:** Brian C. Ross

**Affiliations:** Department of Physics, University of Washington, Seattle, Washington, United States of America; Universiteit Gent, Belgium

## Abstract

Mutual information (MI) is a powerful method for detecting relationships between data sets. There are accurate methods for estimating MI that avoid problems with “binning” when both data sets are discrete or when both data sets are continuous. We present an accurate, non-binning MI estimator for the case of one discrete data set and one continuous data set. This case applies when measuring, for example, the relationship between base sequence and gene expression level, or the effect of a cancer drug on patient survival time. We also show how our method can be adapted to calculate the Jensen–Shannon divergence of two or more data sets.

## Introduction

Mutual information (MI) [1] is in several ways a perfect statistic for measuring the degree of relatedness between data sets. First, MI will detect any sort of relationship between data sets whatsoever, whether it involves the mean values or the variances or higher moments. Second, MI has a straightforward interpretation as the amount of shared information between data sets (measured in, for example, bits); other statistics such as rank-ordering are harder to interpret. Since MI is grounded in information theory it has an established base of theoretical tools. Finally, MI is insensitive to the size of the data sets. Whereas a ‘p-value’ test for strict independence can be pushed arbitrarily low by taking a large data set if the variables are even slightly related, MI will simply converge with tight error bounds to a measure of their relatedness.

The MI between two data sets 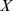 and 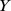 can be estimated from the statistics of the 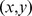 pairs between the two data sets. (Although MI is straightforward to calculate if the underlying probability distribution is known, that is not usually the case: our knowledge of the distribution generally comes from the sampled data itself, so MI must be estimated from the statistics of our data set.) For example, if we were to compare the day of week (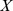) with the time of breakfast (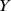) we might find that when 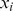 is a weekday the corresponding 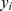 is early in the morning, and when 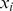 is Sunday or (especially) Saturday the corresponding 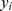 is somewhat later. MI quantifies the strength of this effect. Importantly, the procedure for estimating MI depends on whether 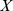 and 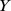 take discrete values (e.g. a day of week, a nucleobase, a phenotypic category, etc.), or are real-valued continuous variables (a time of day, a gene expression level, a patient’s survival time, etc.). If 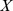 and 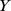 are both discrete, then we can estimate the true frequencies of all combinations of 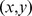 pairs by counting the number of times each pair occurs in the data, and straightforwardly use these frequencies to estimate MI. Real-valued data sets are more difficult to deal with, since they are by definition sparsely sampled: most real numbers will not be found in a data set of any size. The common workaround is to lump the continuous variables into discrete ‘bins’ and then apply a discrete MI estimator, but good sampling requires large bins which destroys resolution. An improved continuous-continuous MI estimator described in Ref. [2] circumvents this tradeoff by using statistics of the spacings between data points and their nearest neighbors. Crucially, their method only works when *both* variables are real-valued, as the nearest neighbor of a discrete variable is not well-defined.

This paper describes a method for estimating the MI between a discrete data set and a continuous (scalar or vector) data set, using a similar approach to that of Ref. [2]. This is an important statistic simply because so many scientific activities involve a search for significant relationships between discrete and continuous variables. For example, one might use MI to quantify the extent to which nationality (a discrete variable) determines income (continuous); to identify DNA bases (ACGT, discrete) that affect a given gene’s expression level (continuous); or to find drugs (given or not: a discrete parameter) that alter cell division rates (continuous data). In the University of Washington Nanopore Physics lab we use this estimator to determine where a given DNA base must sit within the sequencing pore in order to affect the current passing through it, and to quantify the relative influence of different base positions on the current. As we will demonstrate, our nearest-neighbors method estimates MI much more reliably than does the present alternative method of ‘binning’ the data.

MI between a discrete and a continuous variable is equivalent to a weighted form of the Jensen-Shannon (JS) divergence [3] which is used as a measure of the dissimilarity between two or more continuous probability distributions. We can therefore apply our method to estimate the weighted JS divergence, by storing samples from each distribution to be compared in the continuous data set 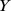, and using the discrete data set 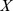 to identify which distribution each sample was drawn from. To use our method to estimate the *unweighted* JS divergence, we would either draw equal numbers of samples from each distribution, or else modify our method somewhat as explained in the Analysis section.

## Methods

This section explains how to apply our nearest-neighbor method for estimating MI; the derivation is left to the Analysis section. We will also describe the binning method that we compare with our estimator.

**Figure 1:**
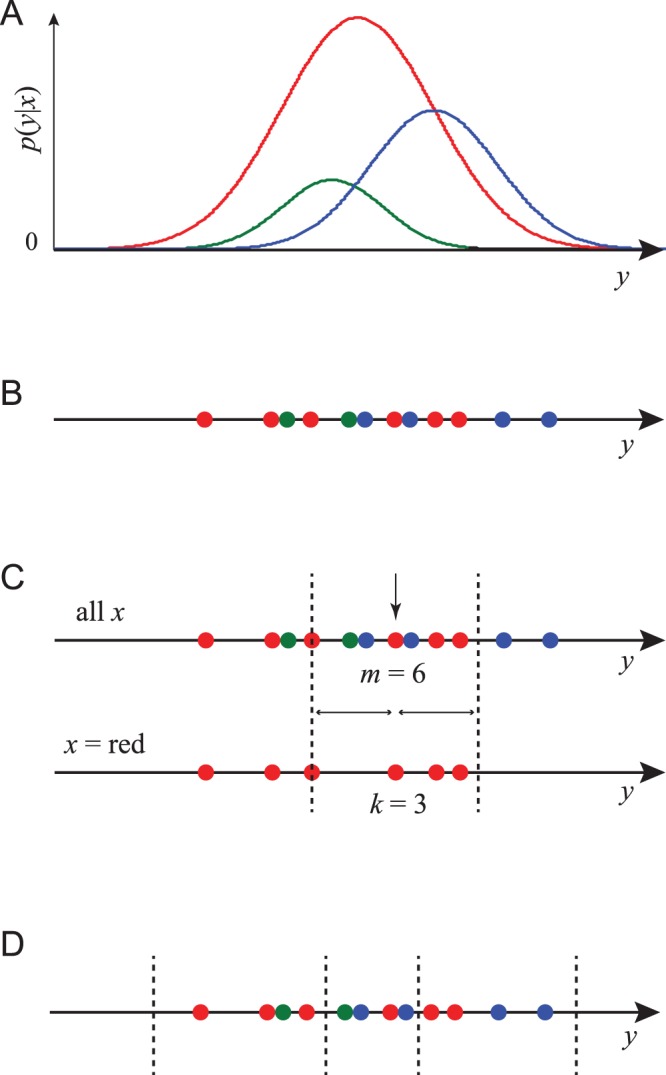
Procedures for estimating MI. (A) An example joint probability density 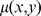 where 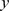 is a real-valued scalar and 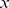 can take one of three values, indicated red, blue and green. For each value of 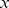 the probability density in 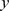 is shown as plot of that color, whose area is proportional to 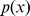. (B) A set of 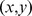 data pairs sampled from this distribution, where 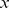 is represented by the color of each point and 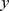 by its position on the 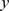-axis. (C) The computation of 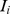 in our nearest-neighbor method. Data point 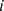 is the red dot indicated by a vertical arrow. The full data set is on the upper line, and the subset of all red data points is on the lower line. We find that the data point which is the 3rd-closest neighbor to 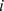 on the bottom line is the 6th-closest neighbor on the top line. Dashed lines show the distance 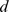 from point 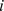 out to the 3rd neighbor. 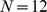, 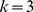, and for this point 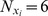 and 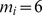. (D) A binning of the data into equal bins containing 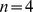 data points. MI can be estimated from the numbers of points of each color in each bin.

The input to a MI estimator is a list of 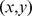 data points, whose underlying probability distribution 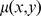 we can only guess at by looking at how the data points are clustered. Both 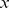 and 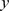 may be either scalars or vectors. Figure 1A illustrates a simple distribution between a discrete parameter 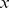 that can take one of three values denoted by color, and a single scalar real-valued variable 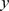 depicted along a y-axis. In this example we see that the different values of 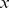 bias the sampling towards different values of 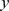: for example 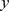 is generally lower when 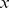 is green or red than when 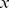 is blue. Therefore there is a relation between 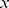 and 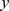, implying that MI is some positive number. The challenge is to estimate MI using only the sampled points that are known to the experimenter (Figure 1B).

### Nearest Neighbor Method

For each data point 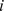 our method computes a number 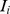 based on its nearest-neighbors in the continuous variable 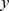, as illustrated for scalar 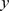 in Figure 1C. We first find the 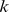
th-closest neighbor to point 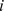 among those 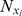 data points whose value of the discrete variable equals 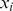 (Figure 1C, bottom line) using some distance metric of our choice. Define 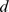 as the distance to this 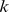
th neighbor. We then count the number of neighbors 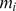 in the full data set (top line) that lie within distance 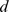 to point 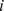 (including the 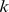
th neighbor itself). Based on 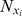 and 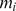 we compute
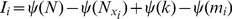
(1)where 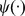 is the digamma function [4]. To estimate the MI from our data set, we average 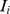 over all data points.
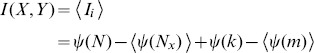
(2)


In our implementation 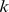 is some fixed (low) integer of the user’s choice; larger 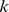-values lead to lower sampling error but higher coarse-graining error.

### Binning Method

We also implemented a binning method to compare with our nearest-neighbor method. Binning methods make the data completely discrete by grouping the data points into bins in the continuous variable 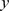, as shown in Figure 1D. Following established practice [2] our estimator constructs bins of different sizes so that each bin has 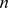 data points inside it (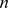 is a parameter set by the user). The binned approximation to the MI is
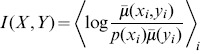


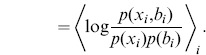
(3)


The average is taken over all measurements 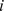, not the bins. 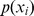 is the fraction of all measurements whose discrete variable is 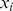, 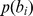 is the fraction of measurements whose continuous variable falls into the same bin 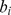 as 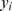, and 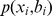 is the fraction of measurements for which 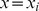 and 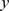 falls into bin 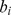. The second line in Eq. 3 follows from the first because we discretize 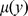 and 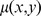 using the same bins.

In the Supporting Information we have included two MATLAB implementations of our method: a general-purpose estimator that works with vector-valued data sets, and a faster implementation for the usual case where both data sets are scalars (simple numbers). The Supporting Information also contains our implementation of a MI estimator using the binning method, as well as the testing script that compares the three estimators and generated the plots for this paper.

## Results

**Figure 2:**
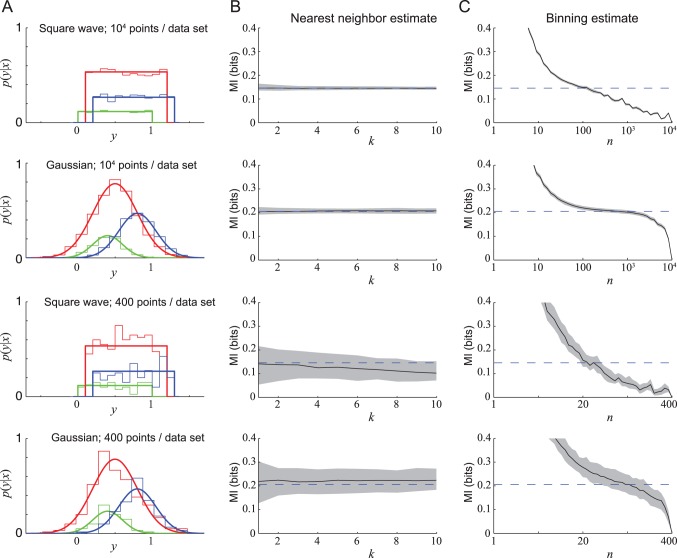
MI estimated by nearest-neighbors versus binning. (A) Sampling distributions 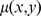 (thick lines) represented by a differently-colored graph in 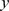 for each of three possible values of the discrete variable 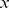 (red, blue and green). A histogram of a representative data set for each distribution is overlaid using a thinner line. (B) MI estimates as a function of 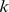 using the nearest-neighbor estimator. 100 data sets were constructed for each distribution, and the MI of each data set was estimated separately for different values of 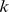. The median MI estimate of the 100 data sets for each 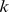-value is shown with a black line; the shaded region indicates the range (lowest 10% to highest 10%) of MI estimates. (C) MI estimates plotted as a function of bin size 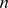 using the binning method (right panel), using the same 100 data sets for each distribution. The black line shows the median MI estimate of the 100 data sets for each 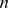-value; the shaded region indicates the 10%–90% range



To test our method, we chose two simple distributions 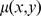: a square wave distribution in 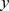 for each value in 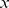, and a Gaussian distribution in 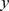 for each 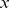 (Figure 2A). Because we knew the exact form of the distributions, we were able to calculate MI exactly using its mathematical definition:

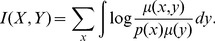
(4)Next, from each distribution, we constructed test data sets by randomly sampling a certain number 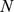 of 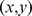 data pairs. We then independently estimated MI from those data sets using our nearest-neighbor estimator and also using our binning estimator, and compared those estimates to each other and to the exact result. We also compared the MI estimate between our vector and scalar implementations of the nearest-neighbor method. Their results in all cases are in exact agreement with each other. This is a strong check that the scripts were written correctly, since the two estimators were coded quite differently.

Both the nearest-neighbor method and the binning method involve a somewhat arbitrary parameter that must be set by the user. The nearest neighbor method requires that the user specify 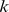 (the 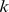
th neighbor). 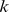 should be some low integer, much less than the number of data points 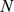, so Figure 2B plots MI estimated by nearest neighbors over the range 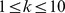. Likewise, the binning method requires that the user specify the number of data points 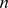 per bin. It is less obvious what the best value of 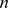 should be; Figure 2C plots MI estimated by binning over all possible values 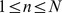.

Our first conclusion is that there is a much simpler prescription for setting the 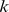 parameter of the nearest-neighbor estimator than the 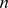 parameter of the binning method. The nearest-neighbor estimator consistently gives good results when 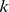 is set to a low integer. Reference [2] suggests using 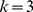, and that choice works well with our estimator too. By contrast, the binning estimator overestimates MI when 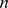 is low and underestimates MI when 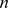 is high, and although there is guaranteed to be a crossing point where the method is accurate it is hard to guess where that point might be. (In the limit 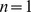 the binning method estimates MI to be the entropy of the discrete variable. The actual MI only attains this maximum limit if the sub-distributions 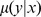 are all completely separated in 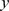. In the limit 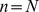 the binning method estimates MI to be zero.).

**Figure 3:**
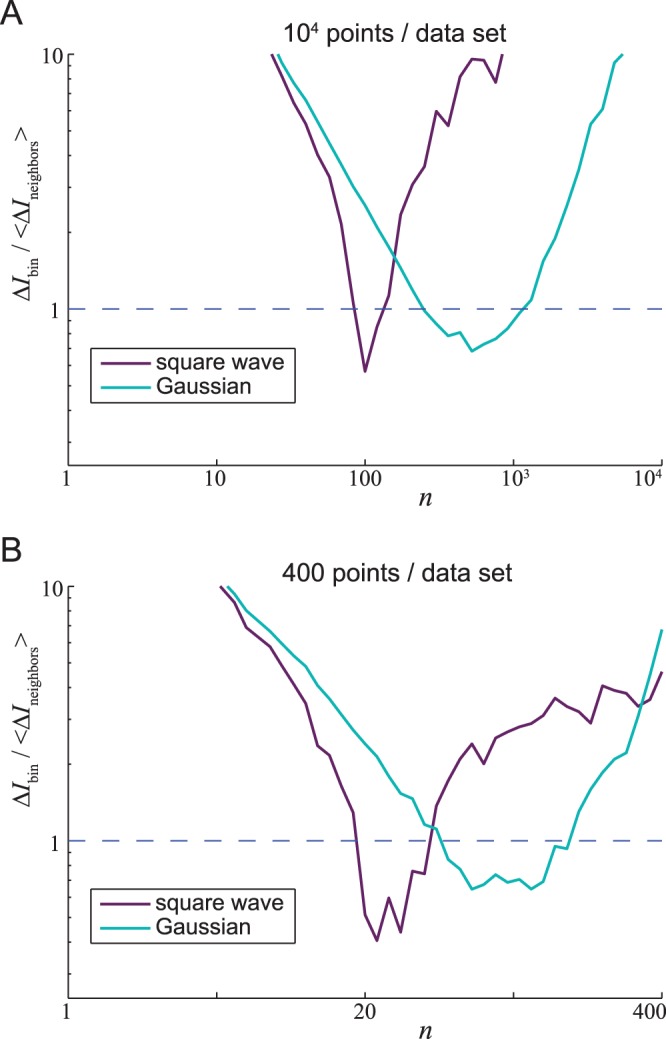
Binning error relative to nearest-neighbors error. (A) Error from the binning method divided by error from the nearest-neighbor method. Errors in MI were calculated for each of the 100 data sets of the square-wave (light blue) and Gaussian (purple) 10,000-length data sets (see Figure 2). Each line shows the ratio of the median MI for a given number of neighbors 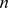 estimated using binning, as a function of *n*, to the median (over all data sets and all values of 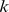) of all MI estimates using nearest neighbors. The binning method gives superior results for values of 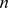 for which this ratio is less than one. Evidently, there is no optimal value of 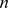 that works for all distributions: 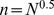 works well for the square wave distribution but 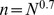 is better for a Gaussian distribution. (B) MI error using nearest-neigbor method versus binning method for the 400-data point sets.

Our second conclusion is that there is *no* simple way to calculate the optimal binning parameter 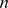 based on simple statistics of the data, such as the total number of data points 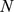 or frequencies with which different discrete symbols occur. For example, the large Gaussian data sets and the large square-wave data sets each have 10000 data points per set, with twice as many red points as blue points on average, and five times more reds than greens. But the best value of 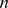 is ∼100 for the square-wave data set and ∼600 for the Gaussian data sets. This is easiest to see in Figure 3A, which plots the ratio of the median binning error using given 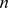 to the median nearest-neighbors error using 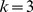. We find that there is no choice of 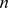 for which binning is better than nearest-neighbors for both the square wave and Gaussian data sets. Figure 3B shows roughly the same result for the 400-point data sets, which again are statistically similar except in the shape of their distributions in 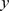.

We conclude that MI estimation by the nearest neighbor method is far more accurate than binning-based MI estimates, barring a lucky guess of the unknowable best value of 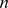. Furthermore, our nearest-neighbor method is computationally cheap: both computation time and memory usage are proportional to 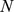 for the scalar estimator. Therefore nearest neighbors should be the method of choice for estimating MI in the discrete-continuous case.

## Analysis

Here we derive the formula for our nearest-neighbor MI estimator.

Consider a discrete variable 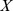 and the continuous variable 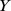, drawn from probability density 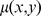. Both 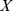 and 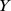 may be either univariate (composed of scalars) or multivariate (vectors). We will write discrete probability functions as 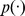 and continuous densities using the symbol 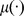: therefore 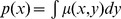 and 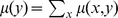. The mutual information is:
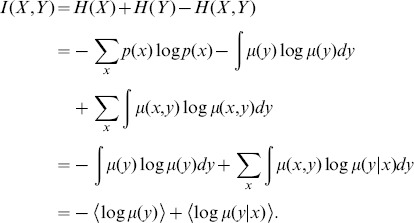
(5)


Here 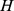 denotes an entropy, 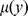 is the probability density for sampling 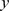 irrespective of the value of 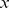, and 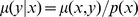 is the probability density for sampling 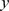 given a particular value of 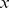. The averages are taken over the full distribution and weighted by 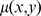, and they would be straightforward to calculate if we knew the underlying density functions. Alternatively, each average can be taken over a representative set from 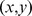 pairs sampled from the distribution; using this latter interpretation we estimate the MI from the mean of 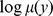 and 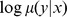 at each of our sampled data points. The more points we have, the greater the accuracy.

The remaining task is to estimate the logarithm of two continuous distributions evaluated at given data points. For this we use a nearest-neighbor entropy estimator originally developed by Kozachenko and Leonenko [5] whose proof we will briefly outline. Given a point 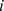, we define 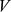 as the volume of points centered about 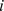 that are closer to point 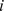 than its 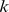
th neighbor. The estimator uses Bayesian arguments to identify 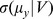 with 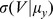 (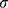 denotes a probability density that is not to be confused with 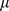). Approximating the density function 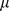 as being constant throughout the neighborhood of point 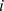, we find:
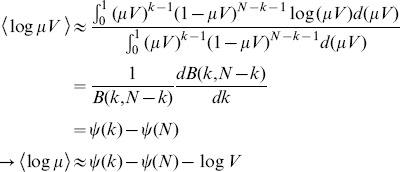
(6)where 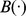 is the beta function [4] and 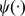 is the digamma function. We can now estimate the entropy using the full data set:
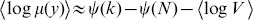
(7)where the average is taken over all sampled data points.

For each sampled data point 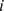 we employ the Kozachenko-Leonenko (KL) entropy estimator twice: once to estimate 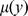 by finding a neighbor from the full set of data points, and once to estimate 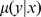 by finding a neighbor in the subset of data points 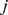 for which 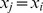. Notice that we can independently choose the neighbors of the two points: we will pick the 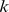
th neighbor in the reduced distribution and the 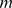
th neighbor from the full distribution. The result is
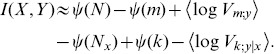
(8)


There is a systematic averaging error that comes from the fact that the 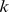
th-neighbor KL entropy estimator applied to point 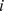 necessarily computes the average of 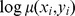 over the volume 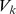, rather than evaluated exactly at point 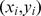. Following Ref. [2], we attempt to minimize this error by choosing 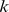 and 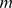 so that both uses of the KL entropy estimator use the same neighbor 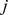. Therefore 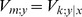 for each data point, and we obtain Eq. 2. The cancellation is only partial; but because the averaging error scales with the number of data pairs as 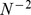 whereas the counting error scales as 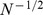, averaging error is generally insignificant except for very small data sets (as we have verified in our tests).

As mentioned before, the mutual information between discrete and continuous data is equivalent to a weighted Jensen-Shannon (JS) divergence between the conditional distributions 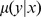, where the frequencies 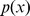 of the discrete symbols 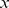 are the weighting factors. To compute an *unweighted* JS divergence we need to place all the conditional distributions on equal footing irrespective of their frequencies in the data, by weighting each term in the averages in Eq. 5 by the factor 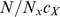 where 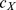 is the number of distinct values that 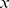 can take. The result is
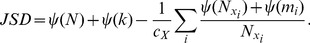
(9)


## Supporting Information

Script S1 **Slow (vector) MI calculator.** Estimates MI between two vector or scalar data sets using the nearest-neighbor method.
(M) Click here for additional data file.

Script S2 **Fast (scalar) MI calculator.** Estimates MI between two *scalar* data sets using the nearest-neighbor method.
(M) Click here for additional data file.

Script S3 **Binning MI calculator.** Estimates MI between two scalar data sets using the binning method.
(M) Click here for additional data file.

Script S4 **Testing script.** Compares the methods using sampled data drawn from user-defined distributions. This script was used to generate the plots in this paper.
(M) Click here for additional data file.
